# A room-temperature refuelable lithium, iodine and air battery

**DOI:** 10.1038/s41598-017-06321-w

**Published:** 2017-07-26

**Authors:** Kim Seng Tan, Andrew C. Grimsdale, Rachid Yazami

**Affiliations:** 1 Energy Research Institute @ NTU (ERI@N), Nanyang Technological University, Research Techno Plaza, X-Frontier Block, 50 Nanyang Drive, Singapore, 637553 Singapore; 2 School of Materials Science and Engineering, Nanyang Technological University, 50 Nanyang Avenue, Singapore, 639798 Singapore

## Abstract

We demonstrate a new refuelable lithium cell using lithium solvated electron solution (Li-SES) as anolyte and iodine solutions as catholyte. This cell shows a high OCV (~3 V). Unlike conventional rechargeable Li batteries, this kind of cell can be re-fueled in several minutes by replacing the spent liquids. We also show for the first time, that Li-SES/I_2_ cells which operate at room temperature, can be prepared in a fully discharged state (~0 V OCV) for safe handling, transportation and storage. Li-SES and iodine are then electrochemically generated during charge as is confirmed by UV-VIS and a qualitative test. We have also conducted proof-of-concept tests for an “indirect lithium-air” cell in which iodine is reduced at the cathode and subsequently is catalytically re-oxidized by oxygen dissolved in the catholyte.

## Introduction

The current focus of research into and development of anode materials for lithium ion batteries (LIBs) is on solid-state anodes such as graphite, carbonaceous materials and carbon-silicon composites^1–5^. Attempts to incorporate both the advantages of the LIB’s high energy density as well as the redox flow batteries’ dual refueling/recharging options, led to the use of alkali-metals (Li or Na) in semi-solid rechargeable lithium flow cells^6–10^. However, these types of cells possess serious disadvantages. Semi-solid flow batteries circulate viscous slurry suspensions from external storage tanks through the cells during operation. The suspensions contain non-conducting lithium-based oxide materials (LiFePO_4_, LiCoO_2_, etc) which require a large quantity of additives to improve their conductivity. Moreover, there is a possibility for solid particles to agglomerate and clog the circulatory system during operation. Present redox-flow cells also have a low energy density. Lastly, solid anode material such as metallic lithium poses a safety concern and must be replaced when depleted.

In this work, we demonstrate a new kind of cell operating at ambient temperature which consists of a liquid-state active anode material (anolyte) and liquid-state active anode material (catholyte) separated by a solid-state Li-ion conducting glass ceramic membrane. Lithium Solvated Electron Solution (Li-SES) is the active material in the anolyte. There are several novel aspects offered by this new chemistry:

1. A low operation OCV of Li-SES versus metallic lithium (<600 mV).
2. High OCV achieved with Li-SES based anode in full cells (>3 V).
3. Cells can be prepared either in the charged state (>3 V), complete discharged state at ~0 V OCV or in the intermediary charged state.
4. The cell uses true liquid solutions which is different from semi-solid flow energy storage devices that utilize slurry-based suspensions of solids as catholyte and anolyte, thus eliminating the issue of particle agglomeration^8, 10^.
5. The depleted cell can be recharged in a variety of methods, one of which is by replacing the spent liquids with fresh ones. This allows the cell to be replenished in minutes, i.e. just like refuelling of cars at a petrol station.

In the initial discharged state, the cell does not have any solvated electrons and iodine to begin with and can be charged to electrochemically produce solvated electrons in the anode and iodine in the cathode as demonstrated in this work.

The LiSES anolyte solution has two main advantages as compared to a solid-state anode or a slurry based anolyte. Firstly, it possesses fast ion transport capability. Secondly, it is able to achieve a physically stable anolyte/ceramic membrane electrolyte interface.

The Iodine/Iodide mixture is dissolved in methanol to form a catholyte solution which can serve as the active material at the cathode^11^ Similar to the anolyte, the catholyte is able to achieve a fast ion transport and stable catholyte/ceramic membrane electrolyte interface.

Our experimental work details the preparation and testing of a charged full cell, a discharged full cell and the FTIR and UV-VIS analysis of anolyte and catholyte solutions, the results of which will be discussed. Also, we introduce the concept of indirect lithium-air cells.

## Results and Discussion

This section is divided into five parts. Part I describes the preparation of anolyte solutions for the charged cell. Part II describes the OCV measurement and discharge profile of a Li-SES//I_2_ full cell loaded in the charged state^11^. Part III describes the CV measurement of an uncharged full cell without the initial presence of both Li-SES in the anolyte and I_2_ in the catholyte, the subsequent generation of the Li-SES and I_2_ from the LiI in both anolyte and catholyte via cyclic voltammetry and finally a qualitative test to check for the presence of Li-SES by dropping a small quantity of the solution in dilute HCl. Part IV covers the FTIR and UV/Vis test results on the anolyte and the catholyte before and after CV charging to verify the formation of Li-SES and iodine. Finally, Part V presents the concept of an indirect Li-Air cell by exposing the catholyte chamber to oxygen.

### Part I: Anolyte Preparation

The LiSES anolyte is prepared chemically for the cell in the charged state.

The LiSES for the charged cell is ${{\rm{Li}}}_{1.0}\beta {({\rm{THF}})}_{12.3}$, where *β* denotes biphenyl and Li: *β*:THF denotes a molar ratio of 1:1:12.3. For clarity, the LiSES will also be referred to subsequently as a solution of 1 M LiSES.

As discussed in our previous study^12^, ${{\rm{Li}}}_{x}\beta {({\rm{THF}})}_{{n}_{1}}$ can be formed in a two-step process according to *x* value of Li. With *x* < 1, ${{\rm{Li}}}_{1}\beta {({\rm{THF}})}_{{n}_{1}}$ forms first together with unlithiated $\beta {({\rm{THF}})}_{{n}_{2}}$:

1 $$x{\rm{Li}}+\beta +n{\rm{THF}}\rightleftharpoons x{{\rm{Li}}}_{1}\beta {({\rm{THF}})}_{{n}_{1}}+(1-x)\beta {({\rm{THF}})}_{{n}_{2}}$$

With *x* > 1, the system consists of a mixture of ${{\rm{Li}}}_{1}\beta {({\rm{THF}})}_{{n}_{1}\text{'}}$ and ${{\rm{Li}}}_{2}\beta {({\rm{THF}})}_{{n}_{2}\text{'}}$:

2 $$x{\rm{Li}}+\beta +n^{\prime} {\rm{THF}}\rightleftharpoons (2-x){{\rm{Li}}}_{1}\beta {({\rm{THF}})}_{{n}_{1}^{^{\prime} }}+(x-1){{\rm{Li}}}_{2}\beta {({\rm{THF}})}_{{n}_{2}^{^{\prime} }},$$

where 1 < *x* < 2.

Separately, a Li-SES solution of the molar composition ${{\rm{Li}}}_{2.0}\beta {({\rm{THF}})}_{22.2}$ is also prepared chemically for FTIR and UV-VIS analysis as well as for a qualitative test using dilute HCl. The results will be covered in **Part IV**. The reader can refer to the supplementary video clip to see the reactions described above.

### Part II: Chemically Prepared Cell

**Figure 1:**
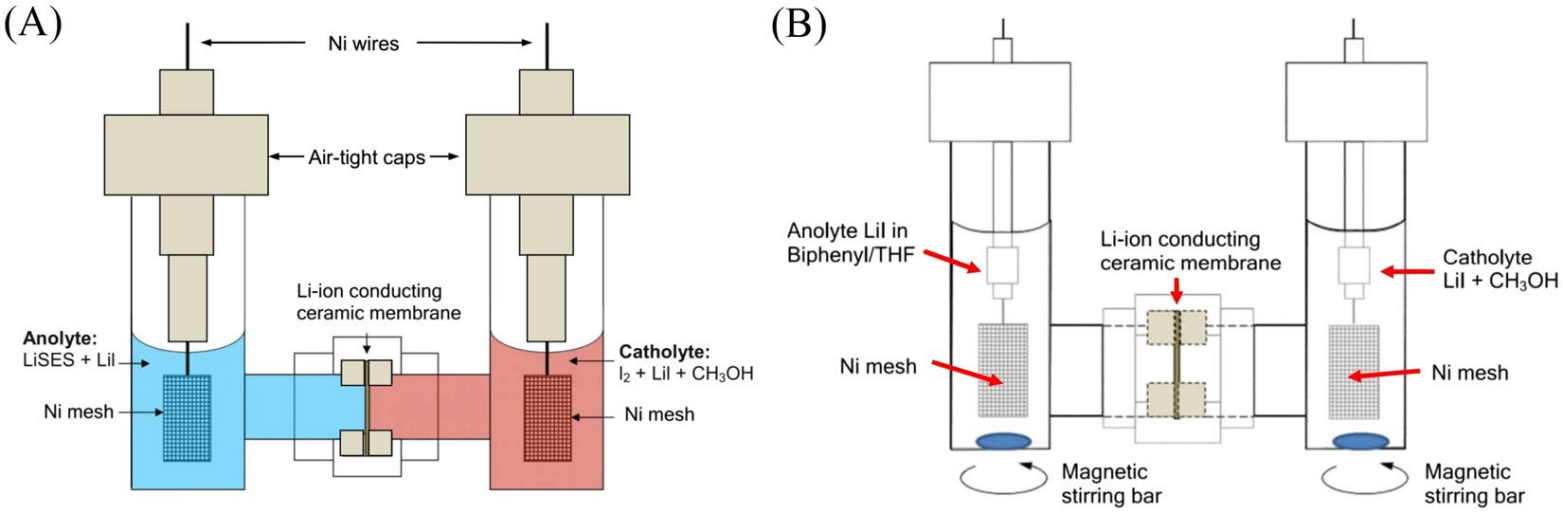
(**A**) Schematic diagram of cell in 100% state-of-charge (S.O.C.)^11^. (**B**) Schematic diagram of cell in 0% state-of-charge (S.O.C). The entire cell sits on two magnetic stirring plates which keep the stirrer bars in the cell stirring.

A schematic diagram of the cell is shown in Fig. 1A. Details of the cell preparation are covered in the Experimental Methods section. The Li-SES anolyte and the Iodine catholyte are separated by a Li-ion conducting solid electrolyte membrane (Ohara, Japan). The initial OCV with 1 M LiSES anolyte and 0.1 M I_2_ catholyte of the cell just before discharge is 2.83 V. The cell is then discharged across a 1 kΩ resistor. A low concentration of iodine in methanol is used because maximum solubility of I_2_ in CH_3_OH is 0.1 M, beyond which additional iodine in the solution exists in solid form^13^.

**Figure 2:**
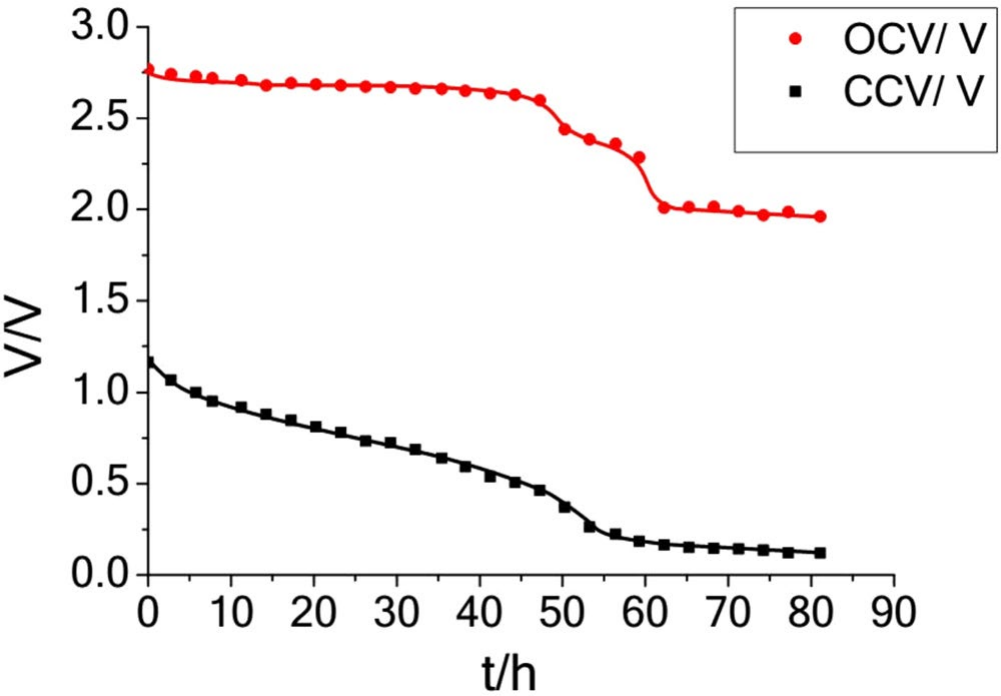
Open-circuit voltage (OCV) and closed-circuit voltage (CCV) profiles^11^.

There is a noticeable drop from 2.83 V to 1.61 V when the cell is first connected to the resistor. This drop could be attributed to the internal resistance of the whole cell in particular in the Ohara thick membrane. Figure 2 shows the open-circuit voltage (OCV) and closed-circuit voltage (CCV) profile of a cell consisting of 1 M LiSES, 0.1 M of I_2_ in methanol and 0.1 M of LiI in both anolyte and catholyte.

Each OCV reading was obtained by disconnecting the cell from the load during discharge and reconnecting back after ~5 min. Two plateaus can be clearly seen in both the OCV versus t and the V versus t graphs. By the end of the first plateau, the catholyte has become colourless, indicating all the I_2_ (or I_3_^−^) has been converted to I^−^.

The electrochemical reactions taking place during discharge and charge of a Li-SES//I_2_ cell can be schematized as follows:

Anode:

3 $$\text{Li}{\rm{-}}\text{SES}\,\underset{{\rm{charge}}}{\overset{{\rm{discharge}}}{\rightleftharpoons }}{{\rm{Li}}}^{+}+{{\rm{e}}}^{-}+{\rm{\Theta }}$$

Cathode:

4 $${{\rm{2Li}}}^{+}+{{\rm{2e}}}^{-}+{{\rm{I}}}_{2}\underset{{\rm{charge}}}{\overset{{\rm{discharge}}}{\rightleftharpoons }}{\rm{2LiI}}$$

Overall cell reaction:

5 $$\text{2Li}{\rm{-}}\text{SES}+{{\rm{I}}}_{2}\underset{{\rm{charge}}}{\overset{{\rm{discharge}}}{\rightleftharpoons }}{\rm{2LiI}}+2{\rm{\Theta }}$$

where Θ in Equations (3) and (5) denotes the PAH/solvent solution^11^.

Since iodine can be in the form of I_3_^−^, the cell reaction may also be written as:

5’ $$3{{\rm{Li}}}^{+}+2{e}^{-}+{{{\rm{I}}}_{3}}^{-}\underset{{\rm{charge}}}{\overset{{\rm{discharge}}}{\rightleftharpoons }}3{\rm{LiI}}$$

Though the subject of using iodine as the liquid-based active cathode material in lithium cells have been explored in the past^14–24^, this is the first time iodine in methanol has been used in conjunction with Li-SES anolyte in a liquid-based cell at ambient temperature^11^. The use of Li-SES as an anolyte was proposed by Sammells^25^, who used liquid ammonia as the electron receptor. However, during discharge, liquid ammonia will convert to gas which will lead to a pressure build-up in the cell. Hence, ammonia-based Li-SES cells are not suitable for use at ambient temperatures. In recent years, we patented poly-aromatic hydrocarbons (PAH) as electron receptors for Li-SES^26^. Our recent studies have indicated that Li-SES prepared using simple PAHs such as biphenyl and naphthalene are stable at ambient temperature^27^.

### Part III: Electrochemically Prepared Cell

In the charged state, both the Li-SES and I_2_ are prepared chemically and loaded into the cell, whereas in the uncharged state, the cell is loaded with LiI in both the catholyte and the anolyte which give a ~0 V initial OCV, then charged to form iodine and Li-SES. The rationale for preparing cells in the uncharged state is the convenience and safety of the system. The cell can also be prepared in a partial charged state depending on the needs.

**Figure 3:**
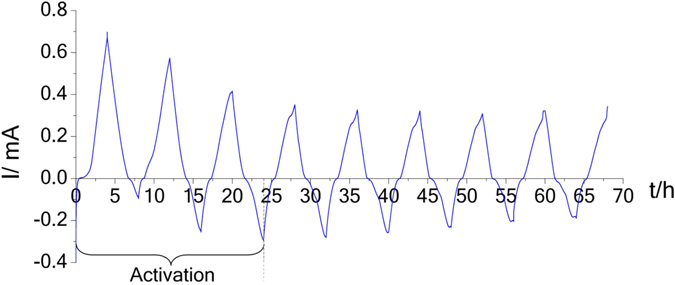
Current vs. time profile during CV cycling (voltage sweeping rate v = 2.5 × 10^−4^ V/s).

A schematic diagram of the uncharged cell is shown in Fig. 1B. The initial OCV of the cell is ~0 V. When the cell is subjected to cyclic voltammetry between 0.8 V and 4.4 V, the colourless catholyte gradually turns dark orange with each cycle, indicating the formation of I_2_ (or I_3_^−^) from I^−^ in methanol according to Eq. (2) (See Supplementary Material Figure S1). The colourless anolyte becomes a very pale amber colour indicating a low concentration Li-SES is formed. With each charge/discharge cycle, the colours of the solutions intensify, suggesting the possibility of some irreversible processes occurring during each cycle. This goes on until the 9^th^ cycle whereby cycling is stopped at 4.4 V. The OCV of the cell is 2.97 V at the end of the 9^th^ charging cycle. The CV profiles are shown in Fig. 3. Earlier studies on electrochemical generation of solvated electrons have shown that solvated electrons that are thus formed are stable at ambient temperature in several types of solvents^28–31^.

From Fig. 3, it can be seen that the cell becomes more stable by the 4^th^ cycle. The current profile during charging after the 3^rd^ cycle (activation) shows change in slope (peak?) followed by steep increase with voltage. The “peak” should be attributed to the I^−^ → I_2_ conversion and the steep increase may come from the liquid electrolyte oxidation (electrolyte wall).

Figure 3 shows the current versus time profile from the CV data. From the areas under each cycle curve, we can determine the amount of charge transferred during each charge/discharge cycle. The Coulombic efficiency during the first cycles is quite low, but increases after the third cycle to above 50%. Hence the first three cycles can be considered as the formation cycles. Integration of the CV data show, at the end of the 9^th^ cycle charge, the Li-SES formed in the anolyte is 7.43 × 10^−3^ M and the I_2_ formed in the catholyte is 3.72 × 10^−3^ M, which is about half of the concentration of the Li-SES formed, as stated by Equation (5).

A direct proof of Li-SES formation is given by the following test: Cell at its charged state up to 4.4 V is removed from the glovebox and a few drops of the anolyte are added to a dilute aqueous HCl solution. The immediate observed evolution of gas bubbles, most likely H_2_ gas, indicates Li-SES is formed in the anolyte solution:

6 $$\text{Li} \mbox{-} \text{SES}+{\rm{HCl}}\to {\rm{LiCl}}+\frac{1}{2}{{\rm{H}}}_{2}$$

The same results have been obtained when a few drops of chemically prepared ${{\rm{Li}}}_{2.0}\beta {({\rm{THF}})}_{22.2}$ is added to dilute HCl.

### Part IV: UV-VIS Analysis of Anolyte and Catholyte solutions before and after CV

**Figure 4:**
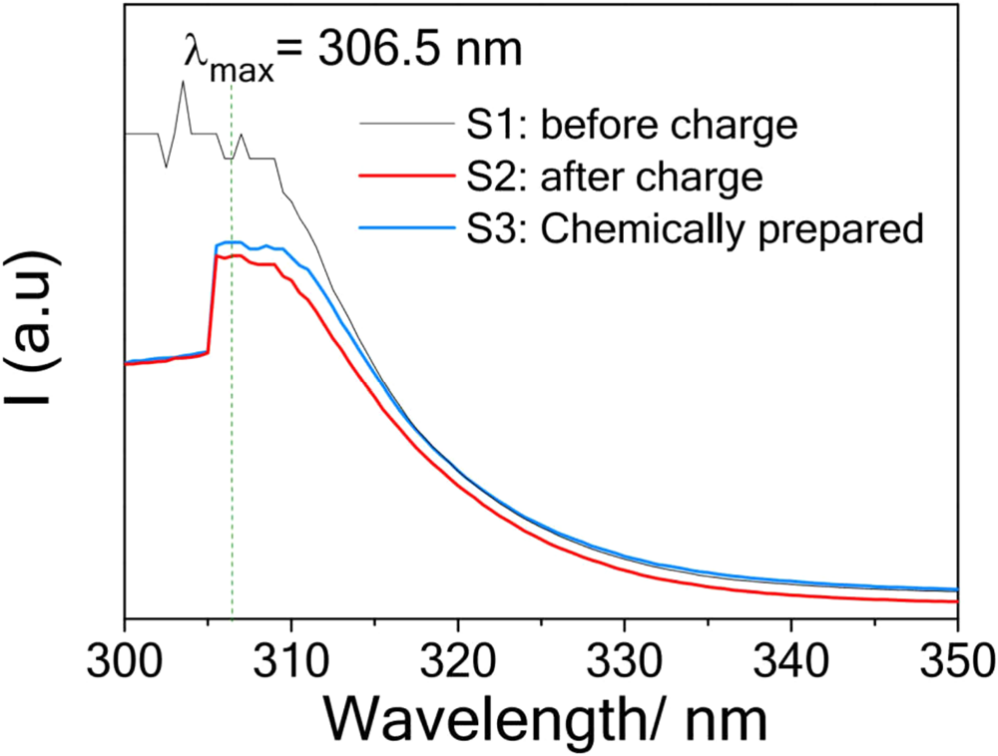
UV-VIS spectra of anolyte solutions of S1 and S2. S1: uncharged anolyte S2: charged anolyte at the 9^th^ cycle S3: Chemically prepared Li-SES.

To provide additional evidence of the electrochemical generation of solvated electrons, UV-VIS spectroscopy measurements are carried out on the anolyte solutions before 1^st^ charging (S1) and after 9^th^ charging to 4.4 V (S2) and are presented in Fig. 4. S3, the spectrum of the chemically prepared Li-SES matches the one of S2. Thus the emerging of the single absorbance peak centred at 306.5 nm in S2 is believed to be caused by the solvated electrons.

**Figure 5:**
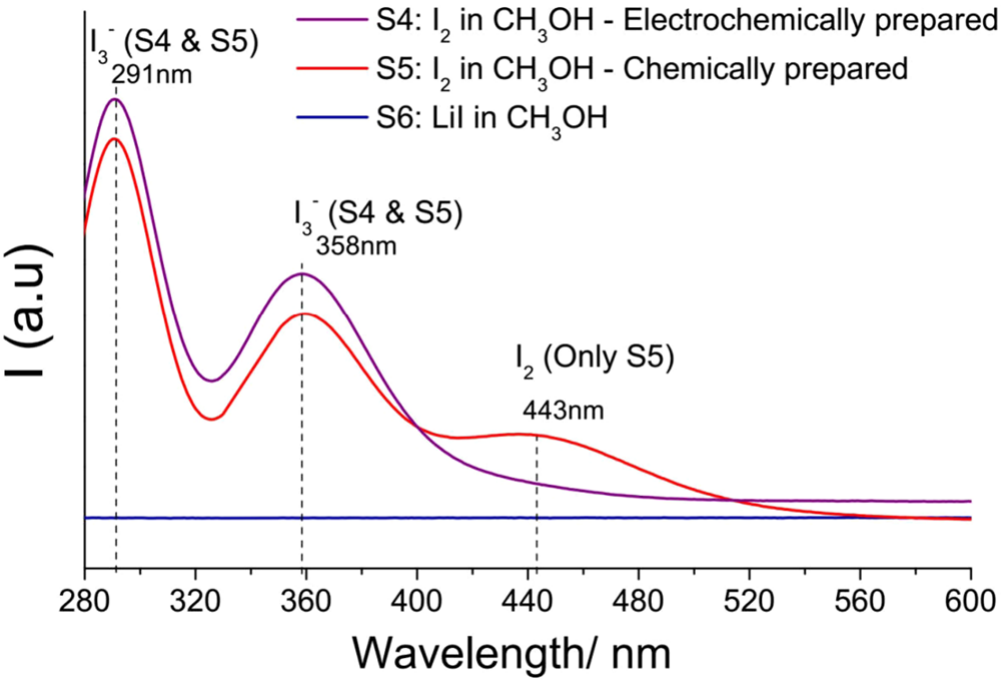
UV-VIS spectra of catholyte solutions before and after charging. S4: Electrochemically prepared iodine in methanol (by charging LiI/CH_3_OH). S5: Chemically prepared iodine in methanol. S6: Lithium Iodide in methanol.

For the catholyte, FT-IR is not able to conclusively distinguish between iodide and iodine unlike UV-VIS, which is able to do so. As presented in the UV-VIS spectra in Fig. 5, S4, (charged catholyte solution at the 6th cycle CV) is different from S6 (lithium iodide dissolved in methanol - uncharged catholyte). In the range of 280 nm to 600 nm studied, spectrum S4 (electrochemically prepared iodine in methanol from charging LiI in methanol) has two peaks at 291 nm and 358 nm in spectrum S4 which are attributed to I_3_^−^. These two peaks matches that of S5 (iodine dissolved in methanol) which indicates that tri-iodide is formed electrochemically via charging^32^. The additional third peak at 443 nm for S5 is attributed to I_2_^33^. In fact, S5 is the characteristic spectrum of iodine dissolved in methanol. The absence of all three peaks (291 nm, 358 nm, and 443 nm) indicates that the LiI/methanol solution consists of only I^-^ in methanol which does not have any peaks in the range studied^33^.

FT-IR analysis has been conducted on the anolyte of the uncharged cell before and after charging and on a chemically prepared ${{\rm{Li}}}_{2.0}\beta {({\rm{THF}})}_{22.2}$ solution as described in **Part I**. However, the amount of solvated electrons formed from our electrochemical process is too small to be detected by FTIR for any meaningful comparison.

### Part V: Indirect Li-Air Cell

A possible further application of iodine-based catholyte is to use it in the presence of oxygen by exposing the catholyte chamber to air during charge/discharge. Our initial tests show that when the catholyte chamber of a chemically prepared cell (Fig. 2) is exposed to air after discharge for a few minutes, the OCV is observed to rise slightly. Also, for an electrochemically prepared cell charged to 4.4 V, its orange catholyte darkened after exposure to air over some time. This colour change suggests the formation of I_2_ perhaps due to oxidation of I^−^ by O_2_. The addition of CuSO_4_ seems to catalyse I^−^ → I_2_ conversion in O_2_ presence because brown solution is formed instantly when CuSO_4_ is added to a solution of colourless LiI in methanol. The I_2_ formed is converted quickly to I_3_^−^ in the solution via the following reaction^34^:

7 $${{\rm{I}}}_{2}+{{\rm{I}}}^{-}\to {{\rm{I}}}_{3}^{-}$$

This is confirmed by UV-VIS (See Spectrum^1^ which has only the two peaks due to I_3_^−^ at 291 nm and 358 nm from Supplementary Material Figure S2). The formation of I_3_^−^ occurs when solid I_2_ is dissolved in methanol as supported also by the UV-VIS spectrum S5 in Fig. 5 described earlier.

A possible reaction mechanism for the catholyte in ‘indirect’ Li-air battery is as follows:

8 $${{\rm{3I}}}_{{2}_{{\rm{solution}}}}+2{e}^{-}\mathop{\longrightarrow }\limits^{{\rm{discharge}}}2{{\rm{I}}}_{{3}_{{\rm{solution}}}}^{-}\quad \quad \quad ({\rm{electrochemical}})$$

9 $$2{{\rm{I}}}_{{3}_{{\rm{solution}}}}^{-}+{{\rm{O}}}_{{2}_{{\rm{solution}}}}\mathop{\longrightarrow }\limits^{{{\rm{CuSO}}}_{4}}{{\rm{3I}}}_{{2}_{{\rm{solution}}}}+{{\rm{O}}}_{{2}_{{\rm{solution}}}}^{2-}\quad ({\rm{chemical}})$$

10a $${{\rm{O}}}_{{2}_{{\rm{solution}}}}^{2-}+2{{\rm{Li}}}_{{\rm{solution}}}^{+}\to {{\rm{Li}}}_{2}{{\rm{O}}}_{{2}_{{\rm{solution}}}}\quad \quad \quad \quad ({\rm{chemical}})$$

Reaction (8) takes place at the cathode current-collector whereas (9) and (10a) occurs in the solution. Even though the catholyte has both I_3_^−^ and O_2_ as oxidants, I_3_^−^ is kinetically more favoured for reduction as compared to O_2_. The Li_2_O_2_ product is formed chemically in the solution as evidenced by the white precipitate at the bottom of the catholyte chamber and not on the cathode as is the case in direct lithium-air cells. Accordingly, the cell reaction in indirect Li-air battery is:

10b $$2{{\rm{LiSES}}}_{{\rm{solution}}}+{{\rm{O}}}_{{2}_{{\rm{solution}}}}\rightleftharpoons {{\rm{Li}}}_{2}{{\rm{O}}}_{{2}_{{\rm{solution}}}}+{\rm{\Theta }}$$

where Θ in (10b) denotes the PAH/solvent solution.

Based on these preliminary results, the cell can be used as an indirect Li-SES/ Air cell in which I_3_^−^ can prevent the formation of passivating Li_2_O_2_ on the cathode current collector during discharge.

## Experimental Methods

### Preparation of Li-SES//I_2_ cell

For the cell set up in the charged state, the liquid-state active anode material (anolyte) is made up of metallic lithium dissolved in a solution of biphenyl in THF. The LiSES anolyte is prepared in its molar composition of ${{\rm{Li}}}_{1.0}\beta {({\rm{THF}})}_{12.3}$, where *β* denotes biphenyl and Li: *β*:THF denotes a molar ratio of 1:1:12.3. The preparation of the Li-SES is carried out in an Ar-filled glove box at ambient temperature. Details of the preparation process are provided in our previous studies^12, 27^. The liquid-state active cathode material (catholyte) is made up of 0.1 M of I_2_ in methanol.

Both the anolyte and the catholyte are separated by a Li-ion conducting ceramic membrane, LATP (Ohara, Japan).

In the uncharged state, the anolyte is made up of a solution of 0.5 M biphenyl with 0.9 M lithium iodide in THF and the catholyte is made up of 0.9 M lithium iodide dissolved in methanol.

In both charged and uncharged cases, lithium iodide serves as supporting electrolyte in both the anolyte and the catholyte.

Lithium foil, iodine, anhydrous methanol, anhydrous THF, biphenyl and copper (II) sulphate were purchased from Sigma Aldrich. Lithium iodide was purchased from Alfa Aesar. All materials were used without further purification.

The completed cells have configurations as follows:

Charged Cell:

(−) Ni mesh/LiSES + THF + LiI//LATP//I_2_ + LiI/Ni mesh (+)

Uncharged Cell (OCV ~0 V)

(−) Ni mesh/ Biphenyl + THF + LiI//LATP//CH_3_OH + LiI/Ni mesh (+)

The plastic components of the cell were machined from PEEK (Polyether-ether-ketone). Both the anolyte and catholyte chambers were made from glass.

### Chemically Prepared Cell

In both anolyte and catholyte, the concentration of the LiI salt (supporting electrolyte) is 0.50 M (Fig. 1A). The initial OCV of the cell is 2.83 V, which is close to the expected value of 2.89 V. This is because the standard electrode potential of biphenyl-based Li-SES is 0.68 V versus Li^35^ and that of I_2_/I^−^ redox couple is close to 3.57 V versus Li.

### Electrochemically Prepared Cell

For this experiment, the anolyte used is 0.9 M of lithium iodide in a solution of 0.5 M Biphenyl in THF (Fig. 1B). The catholyte used is 0.9 M of lithium iodide dissolved in methanol. 25 ml of each solution is used for the cell. Initially, both anolyte and catholyte are colourless. Cyclic voltammetry is conducted on the cell in argon atmosphere. Glass coated magnetic stirrers are placed in both anolyte and catholyte chambers to aid the mass transport of the ions in the solutions. The cell is first charged to 4.4 V and then cycled between 0.8 V and 4.4 V for 8 cycles.

### Electrochemical measurements

A ceramic 1 kΩ resistor is used for constant load cell discharge.

An APPA 505 True RMS Multimeter cum data-logger is used to register the data points for the constant load discharge.

A Basytec Battery Tester is used for the cyclic voltammetry of the initially uncharged cell.

### FTIR and UV-VIS Analysis

An FT-IR spectrophotometer (Perkin-Elmer Spectrum One Spectrometer) with KBr window kit is used. Loading of samples into the KBr window is done in the glove box. After each test, the KBr window is cleaned using Chloroform and dried in a vacuum oven at 80 °C.

UV-VIS spectroscopy measurements are carried out with a Shimadzu UV-2450 spectrometer using a scan speed of 400 nm/min. The baseline correction procedure is executed prior to each measurement session.

### Indirect Li-Air Cell

The catholyte chamber’s cap is unscrewed (loosened) to allow for air to enter the chamber.

CuSO_4_ is added to LiI in air.

## Conclusion

In summary, we have demonstrated for the first time the concept of a liquid cell in the initial charged and discharged states that can operate at ambient temperature. Secondly, we have successful demonstrated the electrochemical formation of Li-SES anolyte and I_2_ catholyte from a discharged cell of 0 V OCV in a full cell configuration. The presence of Li-SES was confirmed by two ways. The first is by qualitative test of adding the charged solution to dilute HCl to produce hydrogen gas. The second way is by UV-VIS which confirms the formation of iodine in the catholyte. This discovery allows us to build a Li-SES//I_2_ cell that has purely liquid based anode and cathode and a solid state electrolyte membrane in either charged or discharged states for refuelable lithium battery applications. Thirdly, we have also introduced iodine in methanol as a solution that can function solely as catholyte. Finally, we have used the iodine catholyte in oxygen to prove the new concept of indirect Li-SES//Air cell.

We are extending our investigations by testing PAHs with more than 2 rings such as 1,3,5-triphenylbenzene derivatives, corranulene^35^ and cyclopenta-2,4-dienone derivatives^36^ as suitable electron receptors for preparing LiSES for use in similar batteries.

A new generation of solid state membranes with enhanced lithium ion conductivity at the ambient temperatures has been developed by us. The new membranes should reduce the ohmic drop in the cell for improved charge/discharge rates^37^.

## Electronic supplementary material

Supplementary materials

Chemical and electrochemical preparation of LiSES, as well as dilute HCl test for LiSES
