# Supplementary material for: A room-temperature refuelable lithium, iodine and air battery
Source: Sci Rep. 2017 Jul 26;7:6502. doi: 10.1038/s41598-017-06321-w (PMC5529521; doi:10.1038/s41598-017-06321-w)
Supplement: Supplementary file 1 — Supplementary materials [file 41598_2017_6321_MOESM1_ESM.pdf]

Supplementary Materials:

A room-temperature refuelable lithium, iodine and air battery

Kim Seng Tan[1,2], Andrew C.Grimsdale[2], Rachid Yazami[1]*

1. *Energy Research Institute @ NTU (ERI@N), Nanyang Technological University,* Research Techno Plaza, X-Frontier Block, 50 Nanyang Drive, Singapore 637553.
2. School of Materials Science and Engineering, *Nanyang Technological University, 50 Nanyang Avenue, Singapore 639798.*

*Corresponding author: Email: rachid@pmail.ntu.edu.sg

Figure S1

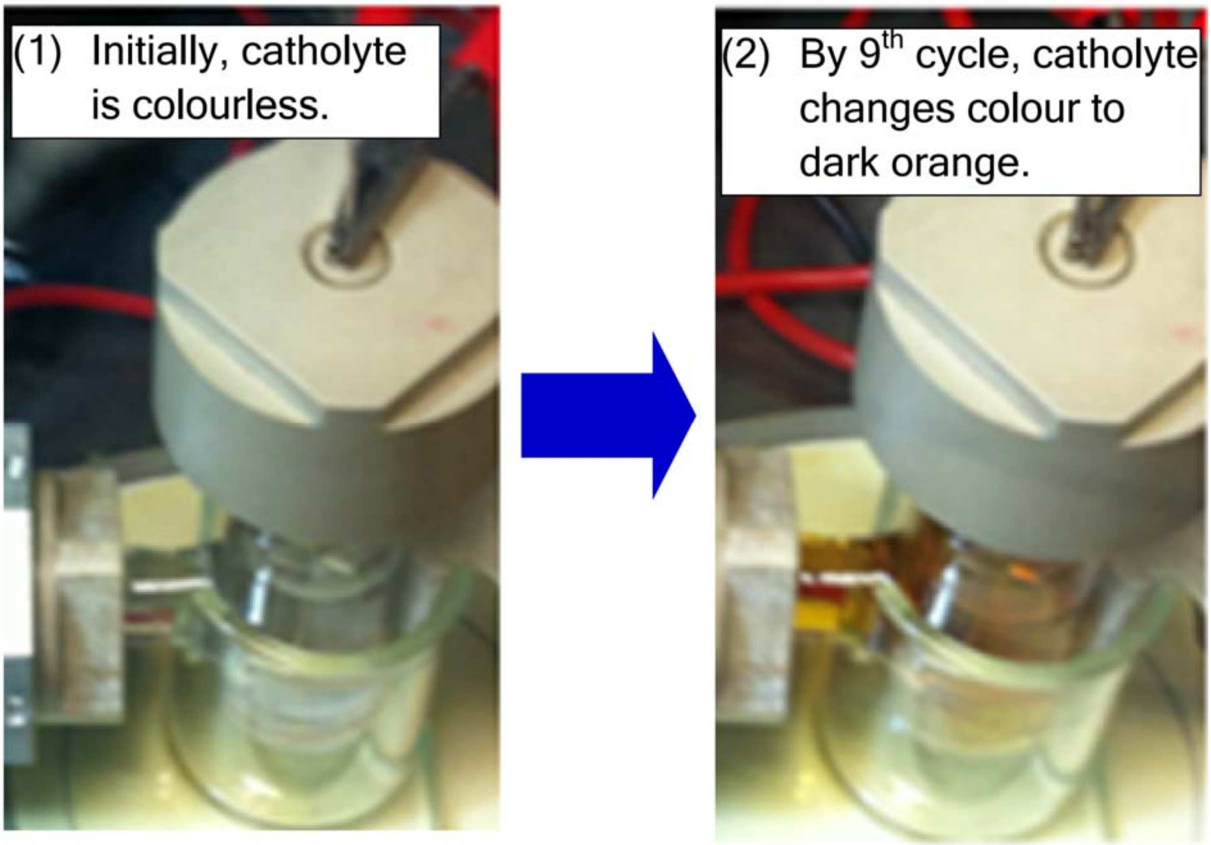

Colour changes taking place in catholyte during the charge-discharge.

Figure S2

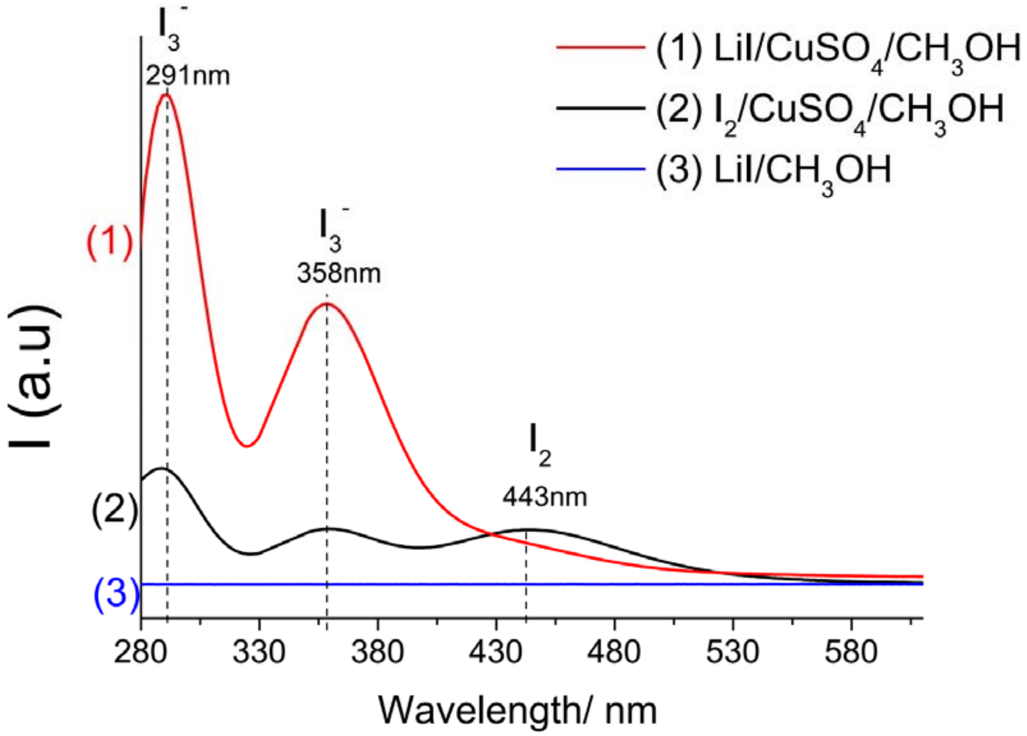

Comparison of (1), (2) and (3) indicates that iodine is present in LiI/$CuSO_4$/CH_3OH

Sample for spectrum (1) is prepared by dissolved 0.1 ~~M/L~~ M LiI and 0.01 ~~M/L~~ M $CuSO_4$ in methanol.

Sample for spectrum (2) is prepared by dissolved 0.1 ~~M/L~~ M I_2 and 0.01 ~~M/L~~ M $CuSO_4$ in methanol.

Sample for spectrum (3) is prepared by dissolved 0.1 ~~M/L~~ M LiI in methanol.

Movie S1 (See attached video file)

Chemical and electrochemical preparation of LiSES, as well as dilute HCl test for LiSES.
